# Screening for Medication Overuse Headache Can Reduce Patients' Suffering From Chronic Daily Headache: A Case Report

**DOI:** 10.7759/cureus.24670

**Published:** 2022-05-02

**Authors:** Maram Alshareef

**Affiliations:** 1 Department of Community Medicine and Pilgrims Health, Umm Al-Qura University, Makkah, SAU

**Keywords:** patient suffering, over the counter, medication overuse, headache, screening

## Abstract

Headache is one of the major global health problems and an economic burden on the population. Common causes of chronic daily headaches are migraine and tension-type headaches, respectively. Medication overuse headache (MOH) is one of the common secondary causes of chronic daily headaches. It appears if the original chronic headache was not treated properly and the patient excessively used over-the-counter medicines as an abortive medication. It can be diagnosed easily if the clinician asks for a detailed history and finds out if the patient fulfills the criteria of MOH. The management requires patient education and withdrawal of the medication use, which can be done successfully most of the time in an outpatient clinic. General practitioners are the initial encounter with this type of patient, so they must screen for this type of headache and establish management to reduce the patient's suffering and burden on other health care facilities.

## Introduction

Headache is one of the common health problems worldwide [1]. It is one of the major burdens on health and economic systems globally [2]. The management approach varies based on the etiology of the headache. Chronic daily headache (CHD) is defined as a daily headache for more than 15 days a month with a duration of ≥4 h over three months [3]. One of the common causes of CHD is chronic migraine, which is defined as headache days for 15 days a month; eight of them had migraine features for the previous three months with a worldwide prevalence of 8.7% yearly. Another cause of CDH is a tension-type headache, with an incidence of 4.7%. It is characterized by daily, dull, aching pain with/without neck or temple tenderness that worsens at the end of the day [4]. One of the secondary causes of CDH is medication overuse headache (MOH). It can result from the misdiagnosis of the original headache or the frequent use of over-the-counter (OTC) analgesics through self-prescription or doctor prescription. It is defined as a headache due to the intake of single or combined analgesics for 10-15 days depending on the type; the worldwide prevalence of MOH is 0.5%-2.6% [5].

The pathophysiology of MOH development is complicated, but it can be a secondary cause of untreated primary chronic headaches [5]. The use of OTC drugs has a risk of renal and liver toxicity in addition to MOHs [6]. The prognosis of this type of headache is generally favorable, with the achievement of 50% fewer headache days [5]. The treatment of the underlying cause of chronic headache can be re-evaluated after the treatment of MOH to provide prophylaxis therapy and thus reduce the incidence of MOH [7-8]. General practitioners are the initial contact with 80% of patients with headaches so they can screen patients for daily medication use to reduce the burden of this problem [9].

Herein, the author presents two patients with misdiagnosed MOH despite being seen by different specialists, which prolongs their suffering.

## Case presentation

Case 1

A 62-year-old woman, with migraine headaches for the previous 20 years, complained of daily headaches with mixed features. The headache was triggered by psychological and physical stress, which aggravated upon waking up. It was sensitive to light and sound and was associated with nausea and vomiting. She experienced daily headaches, but the headaches associated with migraine features happened about 10 days per month.

Regarding the history of previous treatment, she was taking topiramate 25 mg but was discontinued because of side effects. She tried amitriptyline 10 mg for three months, but it was discontinued because of inefficacy. She tried pregabalin 75 mg daily for another three months but stopped because of difficulty in getting a prescription. She tried one cycle of 150 IU Botox (Botulinum toxin A) injection with a neurologist but did not show any improvement. She continued taking different OTC medications daily, including Solpadeine, which contains paracetamol 500 mg, codeine phosphate hemihydrate 8 mg, and caffeine 30 mg, two tablets twice daily, and sumatriptan 100 mg once a day. Her past medical history included hypothyroidism and type 2 diabetes, which were controlled by medication. Socially, she is not a smoker. She is widowed and has five children. Psychologically, she had headache-related depression and reduced social life because she stopped hanging out with her family. She was desperate and looking for ways to manage her headache. The examination showed normal vital signs, no giant appearance of temporal arteries on both temples, and her neurological examination was normal.

In summary, this is a case of a 62-year-old woman with a chronic migraine headache that was not managed well, progressing to chronic migraine in addition to MOH. The patient needed a biopsychosocial approach. The first step taken was to explain the causes of her headache, which was the use of OTC analgesics in addition to no therapeutic dose of previous medications trials used for chronic migraine.

**Figure 1:**
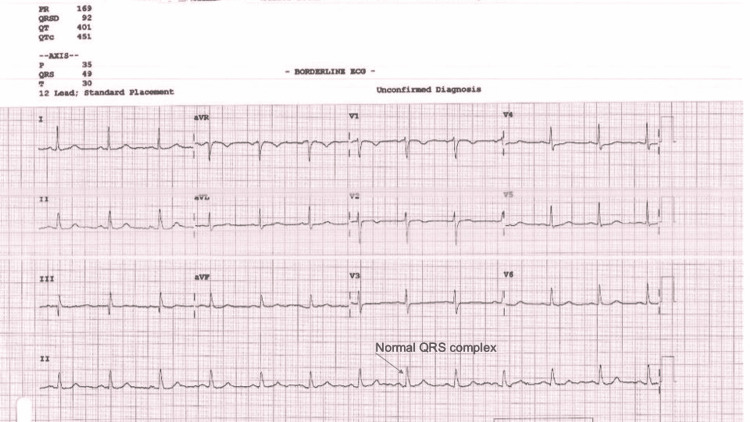
ECG with a normal QRS complex performed for a 62-year-old female with chronic migraine before the initiation of amitriptyline

The treatment for medication overuse was to stop all OTC drugs at once and start preventive medication for headaches. Amitriptyline was chosen because of its effect on CHD and treatment and preventive effects on MOH and migraine [9]. It is also an antidepressant that has mood effects that will improve a patient’s psychological stress and eventually reduce the headache trigger. Electrocardiography (ECG) was conducted before the medications were given (Figure 1).

The medication started with 10 mg and gradually increased up to 40 mg over one month. The patient was instructed to take 10 mg at night and increase the dose to 10 mg weekly until after one month. Regarding breakthrough headaches, the patient was prescribed eletriptan (Relpax) to be taken immediately, as needed, when she feels that a headache is coming but not to exceed 10-12 tablets per month. On the next visit, the patient described having better sleep, but a few times, she needed to take Solpadeine in addition to Relpax, which was taken as instructed. Amitriptyline had a few side effects, such as dry mouth, which was improved by emollients and rehydration. The patient felt a reduction in headache severity, so amitriptyline was increased to 75 mg. The patient returned for follow-up two months after, as instructed, and her headache improved. However, she had used Relpax six times within two months, which was an expected achievement and a great improvement in her headache course over the years.

Case 2

A 51-year-old woman complained of daily headaches for one year. The headache almost always occurred upon waking her and mostly on the right side, with a stabbing nature involving the right temple and area beneath the right eye, associated with lacrimation and rhinorrhea. The pain severity was 10 on a scale of 10 (highest). On her visit to the emergency room, she responded to 100% oxygen flow. The severity and nature of the headaches were the same, as there have been no changes over the year. She has no significant past medical or surgical history.

Regarding social history, she was married and had seven children, all grownups. She had social stressors aggravated by her headaches. She visited a neurologist who prescribed sumatriptan 100 mg daily. On examination, she had normal vital signs, no appearance of giant temporal arteritis on both temples, and her neurological examination was normal. The laboratory results showed normal erythrocyte sedimentation rate (ESR) and complete blood count (CBC). She was diagnosed as a case of MOH on top of chronic cluster headaches based on prolonged symptom duration and described features.

**Figure 2:**
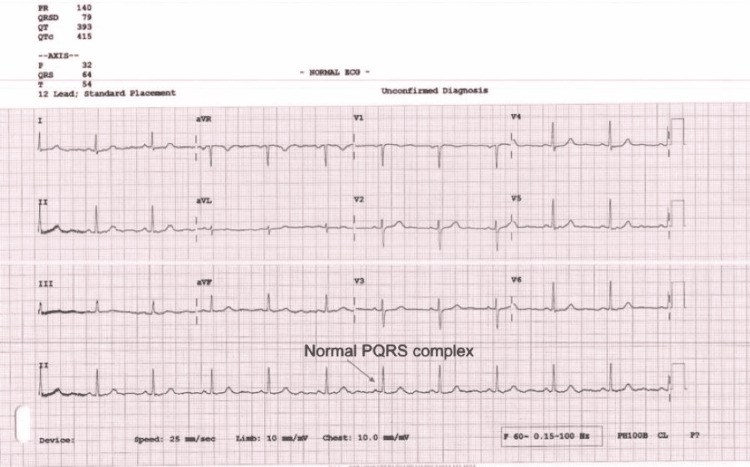
ECG with a normal PQRS complex performed for a 51-year-old female with a chronic cluster headache before the initiation of verapamil

For management, the nature of the headache was explained to the patient, followed by the management plan, which included cessation of sumatriptan, prescription of another headache analgesic, which is a nonsteroidal anti-inflammatory drug or paracetamol, or administration of 100% oxygen flow during an attack, and prescription of preventive medications, such as verapamil with starting dose of 80 mg, to be increased by 80 mg weekly until the headache improved or a dose of 240 mg was reached [4]. A baseline ECG was conducted before the start of drug intake (Figure 2).

The patient was seen one month later; at this time, she reached the full dose of verapamil (240 mg). She still had headaches, but the attacks started to reduce. She was advised to continue the same dose for another two months. Two months later, her headaches improved to three times monthly, and she was responding to paracetamol or ibuprofen. After six months, she was headache-free, and as there were no side effects, she continued verapamil 240 mg for one year, which helped her to regain her quality of life.

## Discussion

Primary headache disorder is common in Saudi Arabia [10]. Moreover, 40% of patients with a headache have CDH [3]. Wrong management of CDH leads to the abuse of OTC analgesics, which subsequently leads to MOH [11]. However, various agents can cause MOH [12]. In the above case scenarios, both patients had a diagnosis of a primary headache disorder but did not receive the proper treatment for it. In addition, none explain to the patient the importance of preventive medication use or the side effects of analgesic overuse. Both patients had headaches for more than three months, localized in the occipital part of the head and starting early morning on waking up. Both used triptan daily for more than three months before their visit [5]. These are indications of MOH. In Saudi Arabia, common OTC analgesics are paracetamol, non-steroidal anti-inflammatory drugs (NSAIDs), and codeine in the form of Solpadeine [13]. Patients and healthcare providers might not take this problem seriously, as these medications are sold OTC, which might give an impression that they are safe for use [14].

However, early screening and diagnosis help patients understand, cooperate, and overcome headaches, which can be achieved through history that analyzes the headache. Furthermore, including a headache diary in the assessment can help clarify the original type of headache and the number of abortive medication overuse attempts and establish the correct diagnosis [9]. MOH is a secondary cause of headaches that can result from a delayed diagnosis, which can complicate the pain and lead to complex types of headaches [8]. Furthermore, this type of headache is treatable and requires counseling of the patient about the OTC drug overuse and the nature of the headache, stopping the medication that was overused, and starting preventive medication. Moreover, 50%-60% of patients with MOH respond to the treatment within three to six months [15].

## Conclusions

Headaches are one of the major causes of economic and health burdens globally. Misdiagnosis of headaches can result from inadequate history during patient encounters, which can be considered malpractice. Unfortunately, this leads to MOH, which can mask the diagnostic criteria and complicate the management of the original headache. Subsequently, the patients suffer from refractory chronic headaches that affect their physical, social, and psychological life. CDH is commonly seen in any general clinic, Hence, the physician must screen and take an adequate history of the headaches, previous treatment trials, number of abortive medications used, and trials of previous preventive therapy. The main step in the management is to confirm the headache diagnosis through detailed history. If the patient fulfills the criteria of MOH, management should be established by educating the patient about the cause of the headache, discontinuing overused medication, and providing correct management based on the type of the original headache.
